# The Dynamic Accumulation Rules of Chemical Components during the Medicine Formation Period of *Angelica sinensis* and Chemometric Classifying Analysis for Different Bolting Times Using ATR-FTIR

**DOI:** 10.3390/molecules28217292

**Published:** 2023-10-27

**Authors:** Fang Ma, Yuan Jiang, Baoshan Li, Yuxin Zeng, Hushan Shang, Fusheng Wang, Zhirong Sun

**Affiliations:** 1 School of Chinese Materia Medica, Beijing University of Chinese Medicine, Beijing 102488, China; 202253008@bucm.edu.cn (F.M.); 20220941431@bucm.edu.cn (Y.J.); 20230935262@bucm.edu.cn (B.L.); 20210935142@bucm.edu.cn (Y.Z.); 2 Dingxi Academy of Agricultural Sciences, Dingxi 743002, China; longhu26@sohu.com (H.S.); 13993208065@126.com (F.W.)

**Keywords:** *Angelica sinensis*, medicine formation period, dynamic accumulation rules, ATR-FTIR, chemometric classifying analysis, quality evaluation

## Abstract

The dried roots of the perennial herb *Angelica sinensis* (Oliv.) Diels (AS) are commonly used as medicinal and edible resources. In commercial planting, early bolting and flowering (EB) of ca. 60% in the medicine formation period reduces root yield and quality, becoming a significant bottleneck in agricultural production. In the cultivation process, summer bolting (SB) occurs from June to July, and autumn bolting (AB) occurs in September. The AB root is often mistaken for the AS root due to its similar morphological characteristics. Few studies have involved whether the root of AB could be used as herbal medicine. This study explored and compared the accumulation dynamics of primary and secondary metabolites in AS and EB roots during the vegetative growth stage (from May to September) by light microscopy, ultraviolet spectrometry, and HPLC methods. Under a microscope, the amount of free starch granules and oil chamber in the AS root increased. On the contrary, they decreased further from EB-Jul to EB-Sep. By comparison, the wall of the xylem vessel was slightly thickened and stacked, and the cell walls of parenchyma and root cortex tissue were thickened in the EB root. Early underground bolting reduces soluble sugar, soluble protein, free amino acids, total C element, total N element, ferulic acid, and ligustilide accumulation, accompanied by the lignification of the root during the vegetative growth stage. Furthermore, a total of 55 root samples from different bolting types of AS root (29 samples), SB root (14 samples), and AB root (12 samples) were collected from Gansu Province during the harvesting period (October). The later the bolting occurred, the less difference there was between unbolted and bolted roots in terms of morphological appearance and efficacy components. Fourier transform infrared spectroscopy with the attenuated total reflection mode (ATR-FTIR) provides a “holistic” spectroscopic fingerprinting of all compositions in the tested sample. The ATR-FTIR spectrum of the AB root was similar to that of the AS root. However, the number and location of absorption peaks in the spectra of SB were different, and only one strong absorption peak at 1021 cm^−1^ was regarded as the characteristic peak of C-O stretching vibration in lignin. The ATR-FTIR spectra can be effectively differentiated based on their various characteristics using orthogonal partial least squares discrimination analysis (OPLS-DA). Results were assessed using multiple statistical techniques, including Spearman’s correlation, principal component analysis (PCA), partial least squares discriminant analysis (PLS-DA), and OPLS-DA. Among these methods, the ATR-FTIR data demonstrated the most effective outcomes in differentiating between viable and non-viable roots for their application in herbal medicine. Essential substances are ferulic acid and flavonoid, which are much more abundant in the AB root. It provides a material basis for the pharmacological action of the AB roots and a theoretical basis for improving their availability.

## 1. Introduction

*Angelica sinensis* (Oliv.) Diels (Umbelliferae) (*A. sinensis*) is a biennial medicinal plant that thrives in cold and damp hillside areas in regions at elevated altitudes between 2200 and 3000 m [1,2]. Min County in the Gansu Province of China is the largest *A. sinensis* production area, accounting for 70% of China’s total production [3]. The dried rhizome of *A. sinensis* (AS) has a long history of use as a hematopoietic and anti-inflammatory agent in traditional herbal medicine. It has been prescribed for treating gynaecological diseases for thousands of years [4,5,6]. These pharmaceutical properties are thought to be primarily associated with bioactive metabolites, including ferulic acid, phthalides, polysaccharides, phenylpropanoids, terpenoids, alkynes, alkaloids, amino acids, trace elements, vitamins, and lipids [7,8,9,10]. Meanwhile, it has found extensive application in the seasoning industry and in the production of tea, health care products, and cosmetics [11]. Consequently, the growing commercial requirements for production and export have expanded the cultivation areas for *A. sinensis*, reaching a current extent of over 43,500 hectares [12].

For commercial planting of *A. sinensis*, seeds are sown in early summer, and germinated seedlings are collected in autumn to be overwintered indoors—the grow-seedling stage. In the following spring, stored seedlings are planted for vegetative growth and harvested in the autumn of this second year for roots, the medicine formation period. We kept the plant in the field until mid-summer of the third year, that is, the reserve-seed period [13,14]. The grow-seedling stage primarily focuses on elongating the root system to fulfil water and nutrient requirements during subsequent growth phases. During the period of medicine formation, the stems and leaves exhibited robust growth, facilitating the accumulation of metabolites. These metabolites were subsequently transported to the roots through the process of photosynthesis. Bolting and flowering are normal physiological processes in plants, essential for seed production and progeny, which are regulated by genetic and environmental factors [15,16,17]. Early bolting of *A. sinensis* (EB) occurred in the medicine formation period and has become a significant bottleneck in agricultural production, and the early bolting rate is up to 60%. During early bolting, large quantities of nutrients in the roots of *A. sinensis* are redirected to the reproductive organs for seed production. Extensive experiments demonstrate that bolting reduces root yield and quality due to the lignification of roots and the degradation of bioactive metabolites. The field investigation found that the premature bolting of *A. sinensis* occurred from June to September. The phenomenon during cultivation primarily refers to summer bolting (SB), which occurs from June to July. The root of SB shows a noticeable difference from the non-bolting source. In addition, autumn bolting (AB) will also happen in the cultivation process in September. Transcriptomics and metabolomics have been employed to investigate the regulatory mechanisms underlying bolting, as well as the reduction in the accumulation of bioactive metabolites and the identification of genes involved in the biosynthesis of ferulic acid, flavonoids, and lignin in *A. sinensis*, which are associated with the shikimic acid and phenylpropanoid pathways [18,19,20,21]. The AB root is often mistaken for the AS root due to its similar morphological characteristics. There are few studies on AB root’s primary and secondary metabolites, which involve whether it could be used as herbal medicine.

The evaluation of *A. sinensis* quality primarily relies on chemical component analysis, which determines the content of single or multiple active ingredients [22]. Secondary metabolites and polysaccharides have been demonstrated as bioactive components to distinguish the different kinds of *A. sinensis* [8]. Nine compounds were selected as distinctive characteristic chemical markers using ultra-high-performance liquid chromatography-quadrupole/time-of-flight mass spectrometry (UHPLC-QTOF-MS/MS) based on metabolomics to rapidly discriminate *A. sinensis* from geo-authentic crude herb and others herb regions. However, the studies mentioned above primarily concentrated on secondary metabolites, neglecting to investigate primary metabolites thoroughly. It is well-recognized that carbohydrates and amino acids are the two significant bioactive chemicals in medicinal/dietary herbs [23,24]. Therefore, to effectively characterize and compare the comprehensive quality of *A. sinensis*, it is essential to consider primary metabolites for adequate overall chemical profiling.

Primary and secondary metabolite accumulation dynamics in the root are closely related to the comprehensive quality evaluation of *A. sinensis*. A study was conducted to investigate the decrease in root biomass in the early bolting plant compared to the unbolting plant, as well as the effects of essential oils at different growth periods [25,26]. The differences in the accumulation rules of ligustilide and phthalides in other medicinal parts of *A. sinensis* from April to October were analyzed by GC-MS. Microscopic identification technology is one of the most practical methods for identifying microstructures, tissues, cells, and subsequent contents [27]. Limited research has been undertaken regarding the identification and microscopic characteristics of the bolting root structure of *A. sinensis*.

The comprehensive evaluation of the superior and inferior quality of medicinal/dietary herbs and the differentiation between them cannot be achieved solely through the analysis of a single chemical component. This limitation arises from various ingredients and multiple targets within herbs. Fourier transform infrared spectroscopy (FTIR), which does not require an invasive or extensive sample preparation procedure combined with appropriate chemometric techniques, is a functional, rapid, additional, or alternative approach for quality control of medicinal/dietary herbs [28,29,30,31]. This approach eliminates the need for invasive or extensive sample preparation procedures and provides a functional, rapid, and potentially alternative analysis means. An infrared spectrum offers a comprehensive spectroscopic analysis that identifies all components in a given sample. The comprehensive spectroscopic fingerprint can effectively demonstrate the presence of bioactive compounds and undesired substances in the tested samples. Thereby, it facilitates the differentiation and identification of *A. sinensis* from other potentially misleading herbs [32]. The operational procedure for sample testing using infrared spectroscopy in the attenuated total reflection (ATR) mode is characterized by its simplicity and rapidity. The products can undergo direct testing without requiring extraction, separation, or other preparation [33,34]. Therefore, it can be concluded that the chemical composition of the tested samples remains unchanged and undamaged. With the availability of software integrating databases, pattern recognition, and calibration models, the quality control of medicinal herbs can be rapidly completed [35,36,37,38].

In the present study, the variation in accumulation patterns and dynamics of primary and secondary metabolites between early bolting root and non-bolting root occurred during the vegetative growth stage (from May to September). The levels of soluble sugar, soluble protein, free amino acids, total C element, total N element, lignin, ferulic acid, and ligustilide contents were analyzed using light microscopy, ultraviolet spectrometry, and HPLC. A one-way analysis of variance (ANOVA) with Duncan’s multiple comparisons was employed to demonstrate the association between the metabolite levels in bolting and non-bolting roots. Furthermore, a total of 55 root samples were collected from various bolting types of AS (29 samples), SB (14 samples), and AB (12 samples) in Gansu Province during the harvesting period in October. Initially, identifying the characteristics and differentiation of the absorption peaks in the ATR-FTIR spectra of three different types of samples was conducted. The quality markers of A. sinensis were analyzed through principal component analysis (PCA) and orthogonal partial least squares-discrimination analysis (OPLS-DA). This study offers a reference for understanding the dynamics of accumulation and evaluating *A. sinensis.*

## 2. Results and Discussion

### 2.1. Microscopic Structure Features of Root Powders during Vegetative Growth Stage

**Figure 1:**
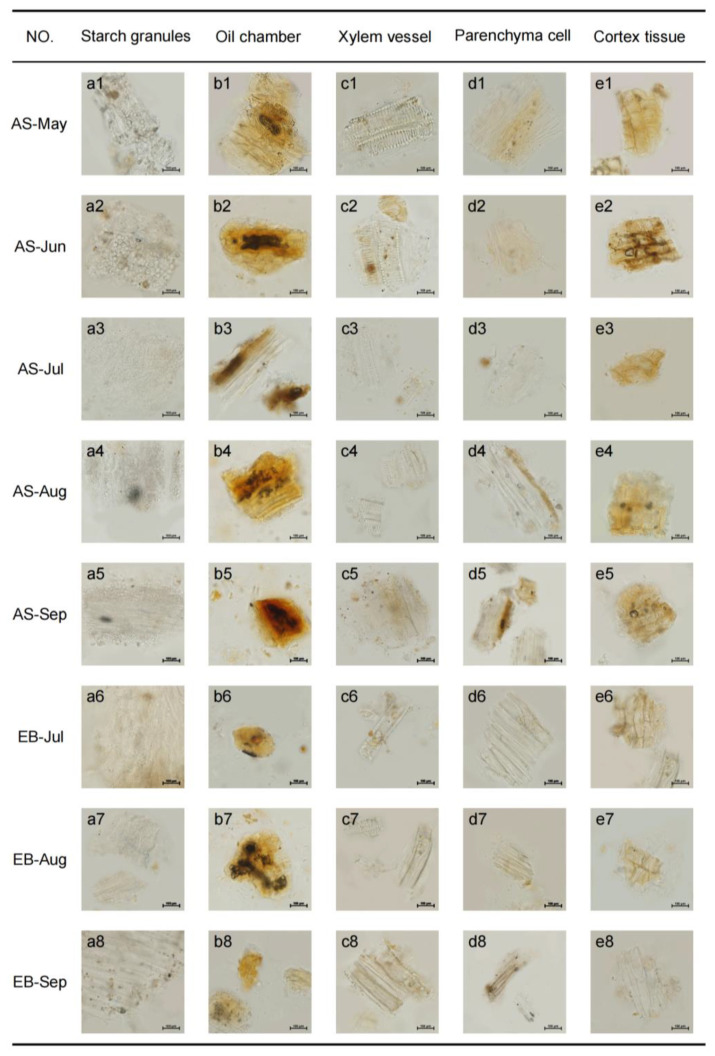
Microscopic features of root powders in different months. (**a**) starch granules, (**b**) oil chamber, (**c**) xylem vessel, (**d**) parenchyma cells, (**e**) cortex tissue (1: AS-May; 2: AS-Jun; 3: AS-Jul; 4: AS-Aug; 5: AS-Sep; 6: EB-Jul; 7: EB-Aug; 8: EB-Sep. Scale is 100 μm.)

The overall organizational morphology structure characteristics change of AS and EB root powders under a microscope were revealed, as shown in Figure 1. Starch is a macromolecular carbohydrate stored in plants. The free starch granules in AS-May powders of the seedling stage are arranged in two to four rows, and most of them exist in parenchyma cells (Figure 1(a1)). The amount of starch gradually increases from the leaf-growing stage to the root enlargement stage (Figure 1(a2–a4)). Clumps of starch granules can be observed inside and out of parenchyma cells in AS-Sep of the late root expansion stage, and the diameter of the object depicted in Figure 1(a5) is 15 μm. On the contrary, the content of starch granules decreased further from EB-Jul to EB-Sep because of bolting (Figure 1(a6–a8)). It is rare to observe clusters of starch granules in EB-Sep powders.

The oil chamber is the organizational structure for storing volatile oils like ligustilide. The debris of the oil chamber structure was observed in AS-May powders, and the secretory cavity contained light yellow oil droplets (Figure 1(b1)). The color of the oil chamber and secretory cavity gradually changes to a deep yellow from June to August (Figure 1(b2–b4)). In September, the diameter of the orange-yellow and fully developed oil chamber structure was observed to be 100 μm. On the other hand, the oil chamber structure and secretory cavity content decreased further from EB-Jul powders to EB-Sep powders (Figure 1(b6–b8)). Only three oil fragments can be found in September.

Terraced and reticulated xylem vessel structures occurred in the early vegetative growth stage (Figure 1(c1–c3)), and the pipeline is comprehensive. A duct bundle is observed in powders due to the root’s enlargement and expansion growth (Figure 1(c4,c5)). In the bolting root powder, the wall of the xylem vessel was slightly thickened and stacked (Figure 1(c6–c8)). The parenchyma cells observed in the unbolted samples exhibited a fusiform shape characterized by thin cell walls. These cells were found to absorb particular oil droplets, and their arrangement was generally loose (Figure 1(d1–d5)). The root cortex tissue wall was mostly quadrangular or hexagonal, with few cell gaps (Figure 1(e1–e5)). By contrast, the cell walls of parenchyma and root cortex tissue exhibited thickening and transformation into elongated fibrous cells (Figure 1(d6–d8,e6–e8)).

### 2.2. Accumulation of Nutrient Composition during Vegetative Growth Stage

**Figure 2:**
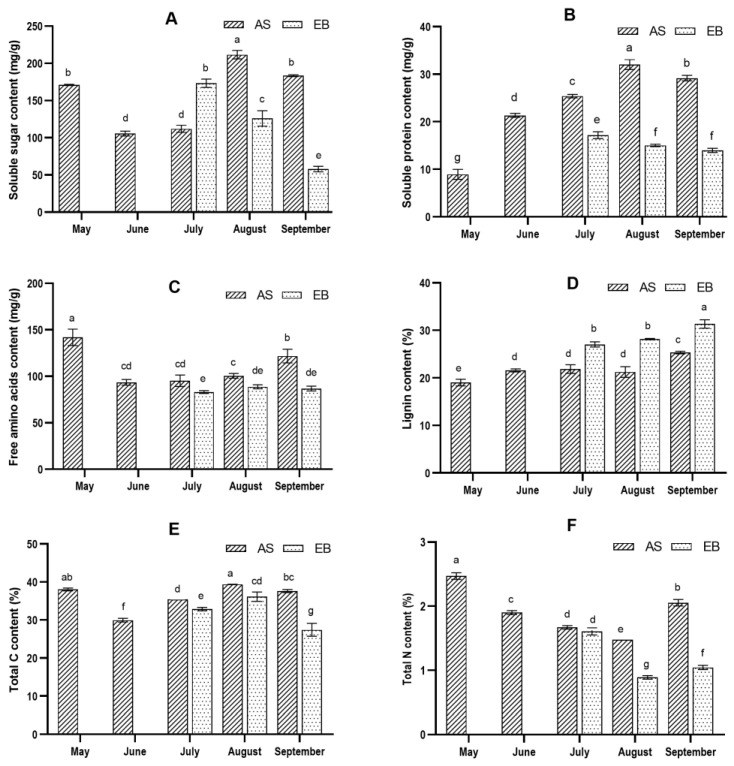
Change in nutritional ingredient content of *A. sinensis* in different months: soluble sugar (**A**), soluble protein (**B**), free amino acids (**C**), lignin (**D**), total C (**E**), and total N (**F**). “Letter a–g” represents a significant difference (*p* < 0.05) in different samples.

The changes in soluble sugar content of AS and EB root collected from May to September are shown in Figure 2A. From AS-May to AS-Jun, the soluble sugar content decreased significantly. The hypothesized explanation is that the polysaccharides stored in the seedlings were utilized for leaf growth. The soluble sugar content increases fast from AS-Jul to AS-Aug, with a difference of 109.56 mg/g in the root enlargement stage. In the later stage of root expansion, the utilization of polysaccharides leads to a decrease in soluble sugar content. Different phenomena occur following premature bolting. The soluble sugar content of EB-Jul was found to be higher compared to AS-Jul. Subsequently, it exhibited a significant decrease, reaching its lowest value of 57.88 mg/g in EB-Sep.

In Figure 2B, the soluble protein content was increased gradually from AS-May to AS-Aug, with the highest value of 32.04 mg/g, along with root growth and enlargement. It fell slightly in September. On the contrary, it was premature bolting that reduced soluble protein accumulation. The content of free amino acids in AS-May had the highest value of 141.93 mg/g (Figure 2C,D). It decreased significantly in AS-Jun due to the aboveground proliferating. Then it increased gradually from AS-Jul to AS-Sep. In July, early bolting resulted in a decrease in the accumulation of free amino acids. The content of free amino acids did not change much until EB-Sep.

The comparison of total C and total N content of AS and EB roots collected from May to September is shown in Figure 2E,F. The observed trend of the two variables exhibited a similar pattern. The carbon content increased overall during leaf growth and root enlargement from June to August. Additionally, the occurrence of early bolting resulted in a higher value. On the contrary, there was a significant decrease in the total nitrogen content during the vegetative growth stage. Additionally, the occurrence of premature bolting resulted in a decrease in the factor mentioned above. In September, a notable disparity was observed in the overall carbon (C) and nitrogen (N) levels.

### 2.3. Bolting Accompanied by Root Lignification during the Vegetative Growth Stage

**Figure 3:**
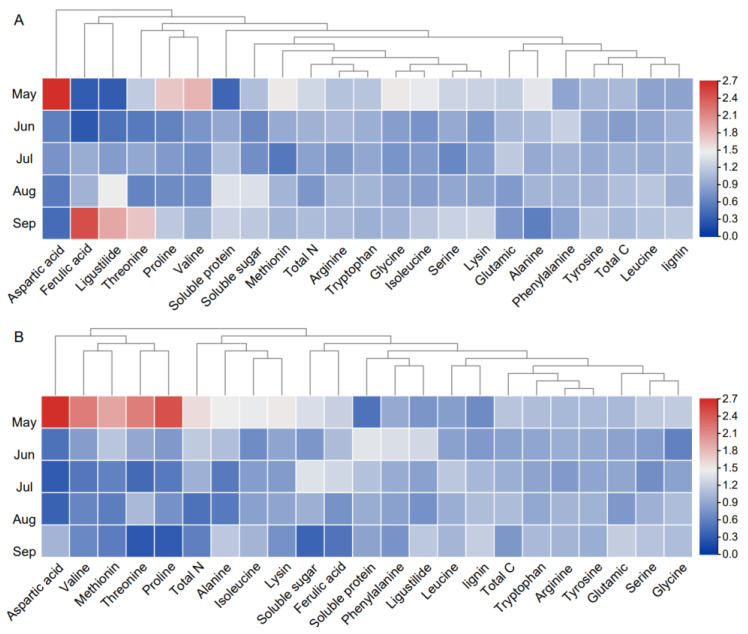
Heatmap of differential metabolites of AS root (**A**) and EB root (**B**) in different months.

As depicted in Figure 2D, the lignin content in EB root powders was higher than in AS root powders. By conducting a comparative analysis of metabolite changes in AS and EB roots, it was observed that various amino acids associated with flowering and root development exhibited differential alterations (Figure 3). Amino acids have multiple functions, including their role as constituents of proteins, as carbon, nitrogen, or sulfur sources, as indicators of cellular nitrogen status, and as precursors of significant metabolites [39]. Leucine and arginine have been implicated in the process of root development [40,41]. The leucine content exhibited a gradual increase in conjunction with the growth and enlargement of the roots from May to September in AS root powder. A notable disparity in EB-Jul was observed after the onset of premature bolting. The arginine content decreased from AS-May to AS-Jul, followed by an increase until AS-Sep. In EB root powders, the addition of early bolting increased the arginine content. Phenylalanine serves as the precursor for the synthesis of anthocyanins, and it plays a crucial role in promoting lignin synthesis [42]. The phenylalanine content exhibited a significant increase from May to June and maintained a higher level. The rapid increase in glutamic and alanine content levels was observed from EB-Aug to EB-Sep, potentially facilitating plants’ flowering and fruiting processes [39]. The observation suggests that the lignification process in the root coincided with premature underground bolting.

### 2.4. Bolting Reduces Ferulic Acid and Ligustilide Content during the Vegetative Growth Stage

**Figure 4:**
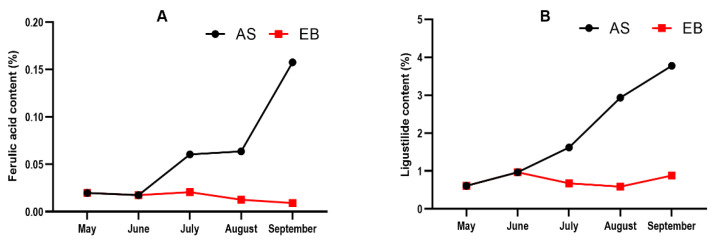
Change in ferulic acid (**A**) and ligustilide (**B**) content of *A. sinensis* in different months.

Ferulic acid content exhibited a 7.78-fold increase at AS-May vs. AS-Sep, with a reduction of 1.11-fold at EB-May vs. EB-Sep (Figure 4A). At the same time, ligustilide content exhibited a 5.32-fold increase at AS-May vs. AS-Sep and a reduction of 0.49-fold at EB-May vs. EB-Sep (Figure 4B).

**Figure 5:**
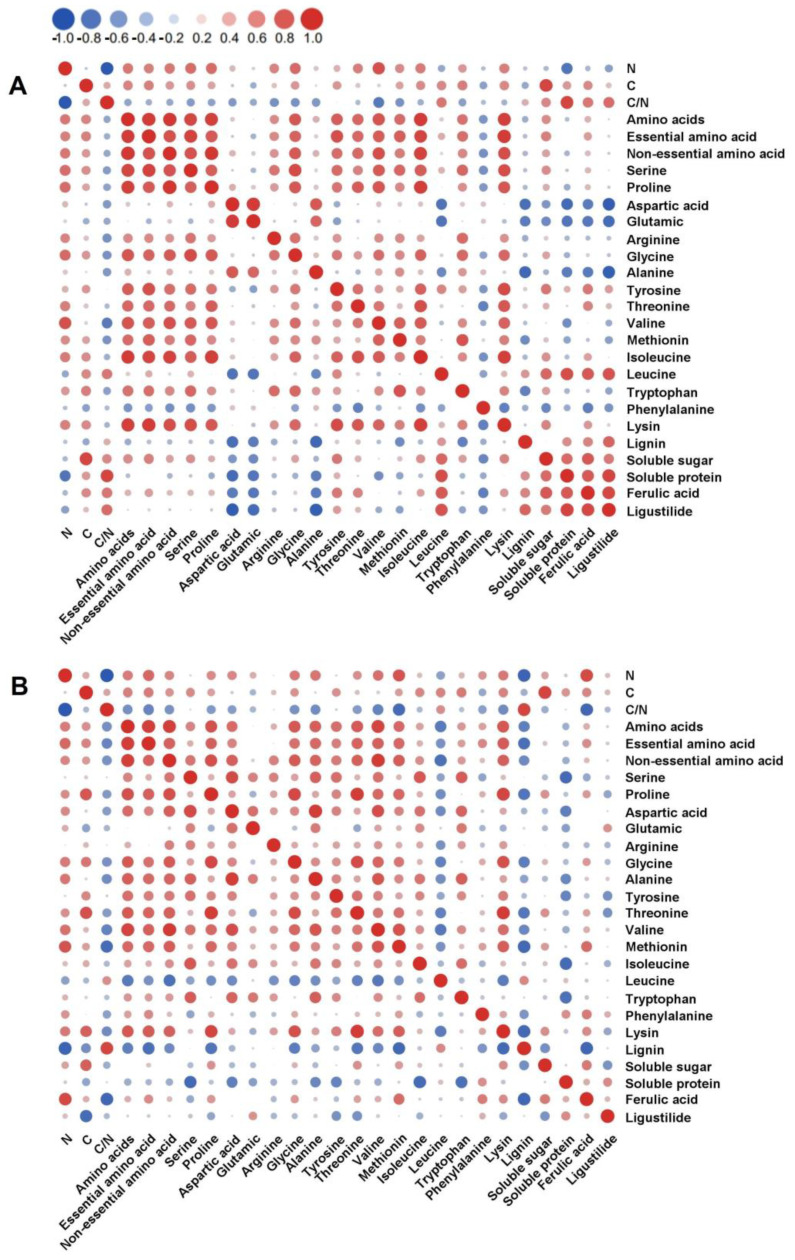
Spearman’s correlations between biochemical indexes of EB root (**A**) and AS root (**B**) in different months. Red and dark-cyan dots indicate positive and negative correlations, respectively; dot color shade indicates the correlation coefficient value.

Spearman’s correlations were conducted to examine the relationship between biochemical indexes of AS root (Figure 5A). The results indicated that ferulic acid and ligustilide exhibited significant positive correlations with soluble protein, soluble sugar, lignin, leucine, and C/N. It was observed that the levels of these compounds exhibited a simultaneous increase during the typical vegetative growth phase. On the contrary, a significant negative correlation was observed between ferulic acid and ligustilide and alanine, glutamic acid, and aspartic acid. The study’s results revealed a decrease in the alanine, glutamic acid, and aspartic acid content during the normal vegetative growth stage. Spearman’s correlations were conducted to examine the relationship between the biochemical indexes of the EB root (Figure 5B). The results indicated a significant negative correlation between lignin and various biochemical parameters, including total N, total C, amino acids, proline, glycine, threonine, valine, methionine, phenylalanine, lysine, soluble sugar, and ferulic acid. The findings suggest that the occurrence of bolting in plants may lead to an increase in lignin content while simultaneously reducing the levels of total N, total C, amino acids (including proline, glycine, threonine, valine, methionine, phenylalanine, and lysine), soluble sugar, and ferulic acid.

### 2.5. Effect of Different Bolting Time on Morphological Characteristics of Root

**Figure 6:**
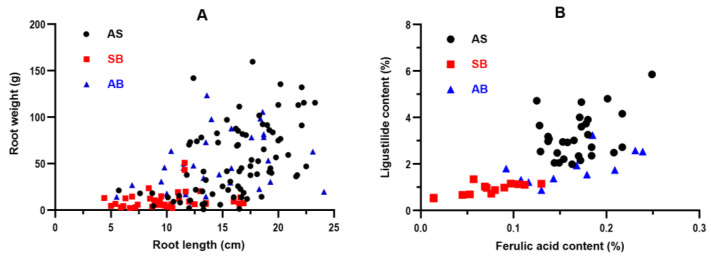
The multiple-variable analysis of root shape (**A**) and active ingredient (**B**) in the harvest period.

The typical morphological appearance and characteristics of the AS root were fleshy with multiple rootlets. The surface was brown, characterized by longitudinal wrinkles and elongated transverse pier-like protrusions. The head of the AS root was slightly cylindrical or irregular in shape. The color and appearance of the AB root were similar to those of the AS root. However, the characteristics of SB roots were different, especially in the number and length of rootlets. There were no longitudinal wrinkles on the surface. The multiple-variable analysis of root shape is shown in Figure 6A. The root length and weight of the AS and EB samples were combined. Although there are differences between SB and others, they cannot be wholly distinguished.

**Figure 7:**
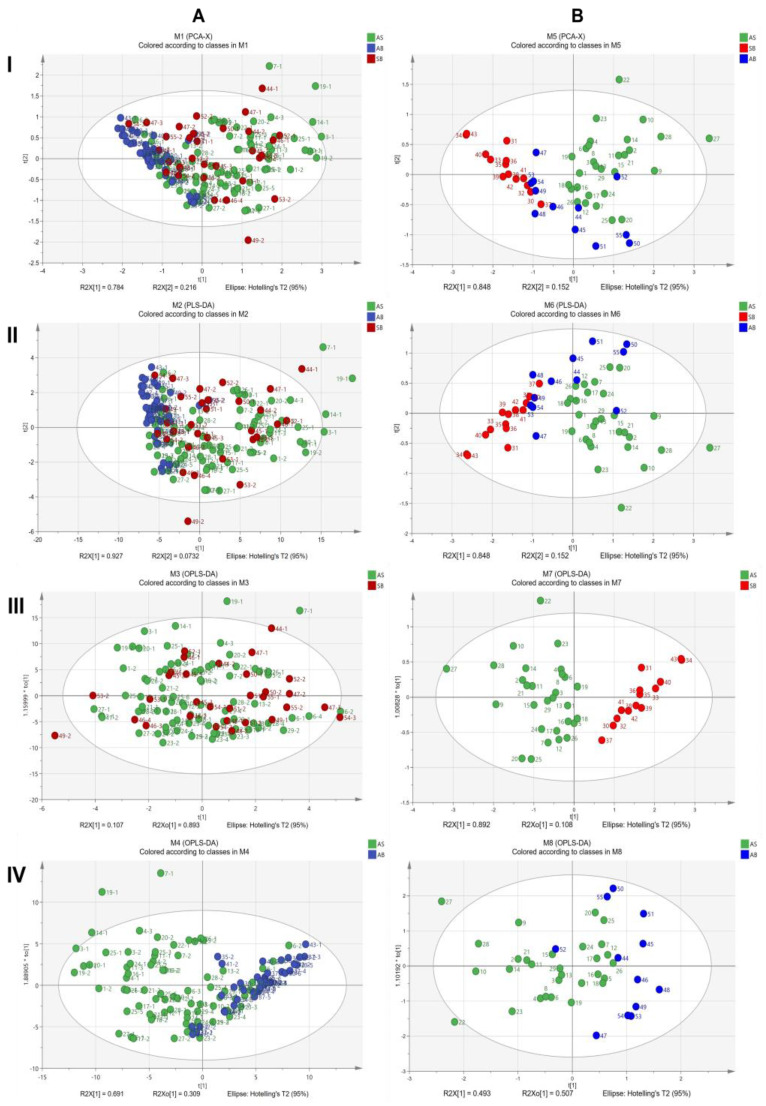
Score scatter plots of root shape (**A**) and active ingredient (**B**) at different bolting times in the harvest period: (**I**) PCA score plots for AB, SB, and AB samples; (**II**) PLS-DA score plots for AB, SB, and AB samples; (**III**) OPLS-DA score plots for AS and SB samples; (**IV**) OPLS-DA score plots for AS and AB samples.

PCA is one of the biometric methods to extract the characteristic features and transform the original correlated variables into a series of uncorrelated variables to create a data set. In Table S2, 29 AS (samples), SB (14 samples), and AB (12 samples) were performed to investigate the morphological characteristics of different bolting times of *A. sinensis* root in the harvest period. The observations included 152 roots, and the variables included root length and weight. Using cross-validation when fitting, with a confidence interval that is 95% by default, the first two PCs accounted for 100% (PC1 = 78.4% and PC2 = 21.6%) of the samples. The PCA score scatter plots showed that the three groups of roots intersected each other (Figure 7A(I)). PLS-DA is a linear pattern classification method extensively employed for handling complex data matrices through dimension reduction. The data underwent processing using the PLS-DA, and the R^2^X (cum), R^2^Y (cum), and Q^2^ (cum) of the data were 1.000, 0.201, and 0.185, respectively. The scatter plots of the PLS-DA scores revealed that the model was not a good fit (Figure 7A(II)). The score plot generated by OPLS-DA analysis did not show a clear separation between the two groups of samples (Figure 7A(III,IV)). The findings suggest that the morphological features of root characteristics do not serve as reliable indicators for distinguishing bolting time, as there is no statistically significant difference between them.

### 2.6. Effect of Different Bolting Times on Active Ingredient in Root

The analysis of ferulic acid and ligustilide contents using multiple variables is depicted in Figure 6B. The AS root exhibited a higher overall level than the other roots. The concentration of ferulic acid in AS reached a maximum of 0.249%, nearly five times higher than the minimum requirement of 0.050% set by the pharmacopoeia. The SB root exhibited the lowest overall level. Nearly 50% of the SB root samples failed to meet the pharmacopoeial requirements. In contrast, ferulic acid and ligustilide content in the AB root were relatively dispersed in the AS and SB roots. PCA and PLS-DA analyses were conducted to examine the active ingredient in A. sinensis root at various bolting times during harvest. The study encompassed 55 samples, as indicated in Table S2. The variables under investigation were ferulic acid and ligustilide. When fitting the data using cross-validation, the first two PCs accounted for 100% (PC1 = 84.8% and PC2 = 15.2%) of the variance. The confidence interval used was 95% by default. The scatter plots of PCA scores revealed a notable distinction between AS and SB roots, with AB roots exhibiting an intersection (Figure 7B(I)). The PLS-DA model’s cumulative R^2^X, R^2^Y, and Q^2^ values were 1.000, 0.459, and 0.453, respectively. The scatter plots of the partial least squares discriminant analysis (PLS-DA) revealed that the model does not fully predict the performance (Figure 7B(II)) between AB roots and the other two samples. The score plot between AS and SB samples in the OPLS-DA analysis (Figure 7B(III)) demonstrates effective separation, whereas the AS and AB samples do not exhibit clear separation (Figure 7B(IV)). The findings suggest that the root’s active ingredient does not completely differentiate the bolting time.

### 2.7. ATR-FTIR Characteristics of Three Types of Roots in the Harvest Period

**Figure 8:**
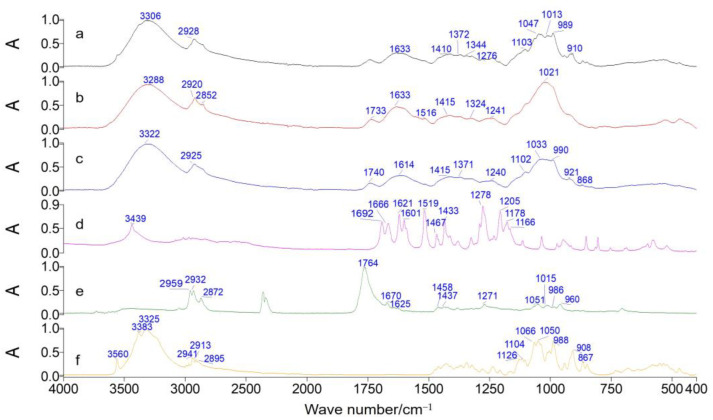
ATR-FTIR spectra of different samples: AS root (**a**), SB root (**b**), AB root (**c**), ferulic acid (**d**), ligustilide (**e**), and sucrose (**f**).

By conducting a comparative analysis of the ATR-FTIR spectra of root powders from AS, SB, and AB, it was observed that the AS root exhibited the highest degree of coincidence, followed by the AB root, while the SB root showed the lowest degree of coincidence (Figure S4). The apparent characteristic absorption peaks of the three types of samples deviated from the range of 1800–1500 cm^−1^ and 1200–800 cm^−1^. The average ATR-FTIR spectra of AS, SB, and AB root powders were individually calculated and compared to standard ferulic acid, ligustilide, and sucrose materials. The outcome is depicted in Figure 8. The absorption peaks observed at 3560, 1103, 1047, 1013, 988, and 868 cm^−1^ in the spectra of AS root (Figure 8a) were found to agree with the peaks observed at 3560, 1104, 1050, 1014, 988, and 867 cm^−1^ in the spectra of sucrose (Figure 8f). These peaks are considered to be characteristic of the O-H bending vibration and the C-O stretching vibration in polysaccharides. The spectral characteristics of the AB root (Figure 8c) exhibited similarities to those of the AS root. However, there were variations in both the quantity and placement of absorption peaks observed in the spectrum of SB root. Only a single firm absorption peak at 1021 cm^−1^ was observed, which is considered to be the characteristic peak associated with the C-O stretching vibration in lignin (Figure 8b). The spectrum of the three types of root powders did not exhibit distinct absorption peaks for ferulic acid and ligustilide.

**Figure 9:**
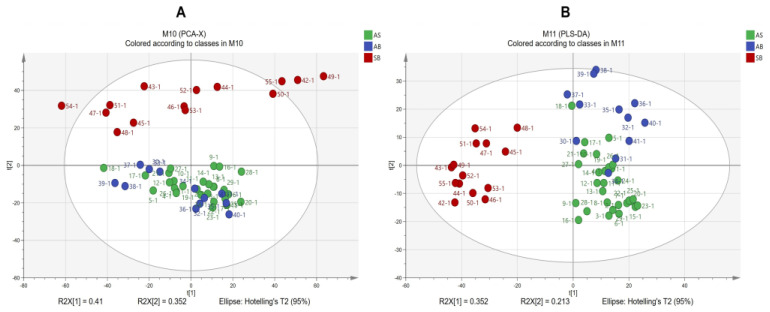
Score scatter plots of ATR-FTIR spectra of root samples in the harvest period: (**A**) PCA score plots for AB, SB, and AB samples; (**B**) PLS-DA score plots for AB, SB, and AB samples.

Based on the integral composition characteristics fingerprint mentioned above, the spectral region of 1800–400 cm^−1^ in the 55 ATR-FTIR spectrum was normalized to conduct chemometrics analysis and distinguish the root of A. sinensis at three different bolting times during the harvest period. The results of the PCA analysis are depicted in Figure 9A. The first two PCs accounted for 75.2% (PC1 = 41.0% and PC2 = 35.2%) of the samples. The samples from the SB root were distinguished from the remaining two samples. The AS and AB root samples belonged to the same cluster. In PLS-DA, the first four principal components were made. The R^2^X (cum) and R^2^Y (cum) of the spectra were 0.905 and 0.823, respectively. The higher R^2^Y value indicates a stronger fit of the model, and the Q^2^ (cum) value of 0.774 suggests good predictability. It is generally accepted that values below 0.9–1.0 indicate excellent predictability. The model validation process utilizes the R^2^ and Q^2^ intercepts acquired during the permutation test. The R^2^ was found to be 0.0922, while the Q^2^ was −0.366. Notably, the values on the left side of the graph were observed to be lower than those on the right side, indicating that the fitted model exhibited predictive capability and accuracy. As depicted in the scatter plots of PLS-DA scores (Figure 9B), the 55 samples were segregated into three distinct groups, with the AS samples falling within a separate category.

**Figure 10:**
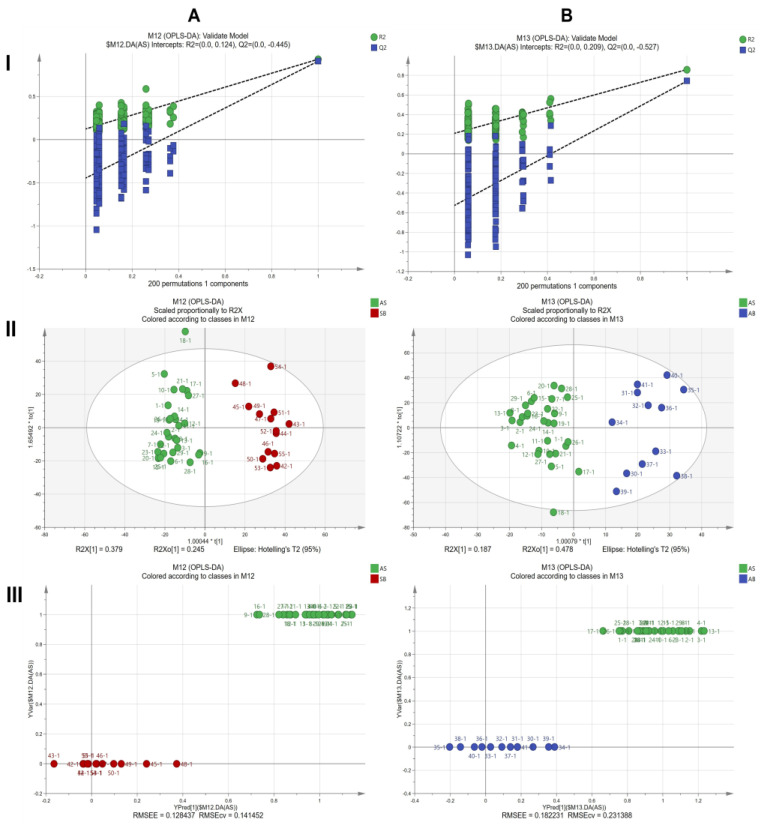
OPLS-DA analysis of ATR-FTIR spectra of AS root samples and SB root samples (**A**), AS root samples, and AB root samples (**B**): (**I**) permutation plot; (**II**) score scatter plot; (**III**) observed vs. predicted plot.

The ATR-FTIR data was processed with the OPLS-DA analysis for the two-class separation. In the OPLS-DA model of AS root samples and SB root samples, the first two principal components were selected, and the result is shown in Figure 10A. R^2^X (cum) was 0.882, R^2^Y (cum) was 0.932, and Q^2^ (cum) was 0.909. The R^2^Y and Q^2^ cross-validation values > 0.9, and the difference between these values < 0.1, indicated that the model has excellent fit and very accurate performance. In the permutation plot (Figure 10A(I)), R^2^ was 0.0124, Q^2^ was −0.445, and the values on the left were lower than those on the right, revealing the predictive fitting model. The score scatter plot (Figure 10A(II)) shows that the AS and SB samples were divided into two clusters. Root mean squared error of estimation (RMSEE) indicates the fit of the observations to the model, which is computed as $\surd (\sum (Yobs-Ypred{)}^{2}÷\left(N-1-A\right))$, where Yobs−Ypred refers to the fitted residuals for the observations at the work site. An alternative predictivity measure for the model available is called the root mean squared error of cross-validation (RMSECV), but it is estimated using the 7-fold cross-validation procedure. In Figure 10A(III), RMSEE and RMSECV were 0.128437 and 0.141452, respectively, indicating that the built model is good. In the OPLS-DA model of AS root samples and AB root samples, the first four principal components were selected, and the result is shown in Figure 10B. R^2^X (cum) was 0.853, R^2^Y (cum) was 0.859, and Q^2^ (cum) was 0.741. The R^2^Y and Q^2^ cross-validation values > 0.7, and the difference between these values < 0.1, indicated that the model has excellent fit and highly accurate performance. In the permutation plot (Figure 10B(I)), R^2^ was 0.0209, Q^2^ was −0.527, and the values on the left were lower than those on the right, revealing that the fitting model was predictive. The scatter plot of the scores (Figure 10B(II)) indicates that the AS and AB samples were segregated into two distinct categories. The RMSEE and the RMSECV were 0.182231 and 0.231388 (Figure 10B(III)), respectively, further used to evaluate whether the OPLS-DA model is accurate. The sensitivity, accuracy, and specificity of the model are 100%. The chemometrics and clustering analysis of the ATR-FTIR spectrum could be used to judge the different bolting times of the *A. sinensis* root.

## 3. Materials and Methods

### 3.1. Plant Materials

#### 3.1.1. Dynamic Materials of *A. sinensis* during the Vegetative Growth Stage

The field trial was conducted in Gufeng village, Gulang County, Wuwei City, Gansu Province, at coordinates 102°43′56″ E and 37°22′32″ N, with an elevation of 2623 m. The soil in question is identified as Hailu soil, with a pH of 7.84. The experimental site possesses a physiological environment that is conducive to the growth of *A. sinensis*, making it a viable location for commercial cultivation. The seedlings were planted in April 2019. The variation of the monthly average maximum temperature and average precipitation in Gufeng village from May to September 2019 is shown in Figure S1 (the meteorological data were from the official website of the China Meteorological Observatory). The collection of plant materials occurred at various stages of growth, namely the seedling stage on 21 May, the leaf growing stage on 19 June, the leaf and root growing stage on 15 July, the root enlargement stage on 17 August, and the late root expansion stage on 20 September. Early bolting of the plant is observed in the latter part of June. Take a sample of 5 to 10 plants on each occasion and replicate the process three times. In addition to the five non-bolting samples (AS-May, AS-Jun, AS-Jul, AS-Aug, and AS-Sep), three samples exhibited early bolting (EB-Jul, EB-Aug, and EB-Sep). All samples were transported back to a refrigerated container.

#### 3.1.2. Different Producing Areas Materials of *A. sinensis* during the Root Harvest Period

A total of 55 samples, consisting of 29 samples of AS, 14 samples of SB, and 12 samples of AB, were collected from Gansu Province during the harvesting period of *A. sinensis* in October 2019. The sample information is displayed in Table S1. The rhizomes of *A. sinensis* were dried until a constant weight was achieved, using a temperature oven set at 60 °C. The dried samples were pulverized and sieved through a 60-mesh sieve for subsequent utilization.

### 3.2. Microscopic Identification of Powdered Crude Root

The vegetative growth stage of *A. sinensis* involved crushing all dried roots into a powdered form using a grinder. The resulting powder was then sieved through a 100-mesh sieve with a bore diameter of 0.150 mm. The crushed samples were permeabilized with chloral hydrate and sealed with diluted glycerin for observation [43,44]. Eight different slides of powdered samples were examined using ordinary light microscopy. Distinctive microscopic features of the powder were also documented.

### 3.3. Determination of Soluble Sugar Content

The glucose reference standards (10.03 mg) were meticulously weighed and dissolved in 100 mL of distilled water to achieve the final 0.1003 mg/mL concentrations. Subsequently, precise volumes of 0 mL, 0.1 mL, 0.2 mL, 0.4 mL, 0.6 mL, 0.8 mL, and 1.0 mL were absorbed and brought to a constant volume of 1 mL with distilled water. Following this, 5 mL of sulfate-anthrone reagent was added. The absorbance at 620 nm was measured by an ultraviolet spectrophotometer.

The dynamic dried *A. sinensis* powder (50 mg) was precisely measured and then added to 10 mL of distilled water. The samples were put in a water bath for 20 min at a temperature of 100 °C. They were centrifuged at 13,000 revolutions per minute for 10 min. The resulting supernatants were carefully transferred into 50 mL volumetric bottles, and the volume was adjusted to the designated scale line. Precisely aliquot 0.2 mL of the liquid and dilute it to a final volume of 1 mL with distilled water. Subsequently, introduce 5 mL of sulfate-anthrone reagent. The absorbance at 620 nm was measured by an ultraviolet spectrophotometer.

### 3.4. Determination of Soluble Protein Content

The reference standard used in this study was bovine serum albumin (Solarbio, Beijing, China) with a concentration of 0.501 mg/mL. Subsequently, precise volumes of 0 mL, 0.1 mL, 0.2 mL, 0.4 mL, 0.6 mL, 0.8 mL, and 1.0 mL were absorbed and brought to a constant volume of 1 mL with distilled water. Finally, 5 mL of Coomassie brilliant blue dyeing solution was added. The absorbance at 595 nm was measured by an ultraviolet spectrophotometer.

The dynamic fresh root of *A. sinensis* (1000 mg) was accurately weighed and then added to 9 mL of phosphate-buffered saline (pH = 7.2–7.4) to make pulp. The supernatants were transferred after being centrifuged at 13,000 rpm for 10 min. Precisely absorb 0.2 mL of the liquid to a constant volume with distilled water to 1 mL, and add 5 mL of Coomassie brilliant blue dyeing solution. The absorbance at 595 nm was measured by an ultraviolet spectrophotometer.

### 3.5. Extraction in the Identification of Free Amino Acids Contents

The dynamic dried *A. sinensis* powder (200 mg) was precisely measured and introduced into 10 mL of distilled water. The samples were put in a water bath for 15 min at 80 °C and then centrifuged at 6000 rpm for 10 min; the resulting supernatants were derived for the following work.

An LC-20AT HPLC system (Shimadzu, Kyoto, Japan) with an RF-10AXL fluorescence detector and LC solution chromatographic data workstation was employed to analyze amino acid constituents in samples. The separation of analytes was achieved on a DIKMA Diamonsil AAA column (5 µm, 250 × 4.6 mm) and maintained at 45 °C. The detection wavelength was 360 nm. The flow rate was 1.0 mL/min, while the sample injection volume was 10 μL. The mobile phase consisted of 0.02% K_2_HPO_4_ + 0.02% KH_2_PO_4_ (A) and 1% acetonitrile in methanol (B), with the following optimized gradient elution: 0–39 min, 14–40% B; 39–44 min, 40–70% B; 44–65 min, 14% B.

The mix of sixteen kinds of free amino acid reference standards was obtained to the final concentrations of 0.065 mg/mL for aspartate, 0.066 mg/mL for glutamic, 0.051 mg/mL for serine, 0.086 mg/mL for arginine, 0.040 mg/mL for glycine, 0.023 mg/mL for threonine, 0.046 mg/mL for proline, 0.032 mg/mL for alanine, 0.022 mg/mL for valine, 0.008 mg/mL for methionine, 0.010 mg/mL for isoleucine, 0.020 mg/mL for leucine, 0.098 mg/mL for tryptophan, 0.020 mg/mL for phenylalanine, 0.075 mg/mL for lysine, and 0.099 mg/mL for tyrosine. The mixed reference standard was derived and sampled precisely at 2 μL, 4 μL, 6 μL, 8 μL, 10 μL, 20 μL, and 30 μL and subjected to HPLC analysis.

### 3.6. Determination of Total C and Total N Element Content

The quantification of total carbon (C) and nitrogen (N) content in dynamic *A. sinensis* was performed by an ECS 4024 CHNSO analyzer (NCT, San Clemente, Italy). CO_2_ and N_2_ were generated from C and N in solid samples in a redox tube at a temperature of 980 °C. The sample (including C, H, and N) was subjected to complete combustion, and a mixed gas of CO_2_, N_2_, and H_2_O was generated. The generated gas was separated in the apparatus equipped with gas chromatographic (GC) columns (70 °C, 2 m). The flow rate of a gas carrier (He:O_2_, 4:3) was 100 mL/min. The TCD detected CO_2_ and N_2_ in the mixed gas. A sorghum flour standard series concentration obtained a calibration curve with 1.47% N content and 41.26% C content.

### 3.7. Determination of Lignin Content

A dry sample weighing 5.0 mg was added to a solution containing 5 mL of 30% acetyl-bromide solution in glacial acetic acid (*v*/*v*) and 0.2 mL of 1 mol/L perchloric acid. The samples were placed in a water bath for 60 min at 70 °C, then added to 10 mL of glacial acetic acid and 10 mL of NaOH (2 mol/L) to terminate the reaction. The liquid had a constant volume of 50 mL of glacial acetic acid. The absorbance of the solution (mixed immediately before the reading) was read in a UV-spectrophotometer at 280 nm against blank samples using quartz cuvettes. A calibration curve was obtained by detecting the absorbance of a series of concentrations of lignin standard solution (0.00215 mg/mL, 0.0043 mg/mL, 0.0198 mg/mL, 0.0240 mg/mL, and 0.0276 mg/mL) at 280 nm after acetylation.

### 3.8. Extraction in the Identification of Ferulic Acid and Ligustilide Contents

The dried *A. sinensis* powder (200 mg) was accurately weighed and then added to 20 mL of methanol. The samples were sonicated for 40 min at 25 °C and centrifuged at 13,000× *g* for 10 min; the supernatants were transferred to liquid injection vials.

An LC-20AT HPLC system (Shimadzu, Japan) with an RF-10AXL fluorescence detector and LC solution chromatographic data workstation was used to analyze the samples’ ferulic acid and ligustilide constituents. The separation of analytes was achieved on a Waters CSH C18-RP column (1.7 µm, 150 × 2.1 mm) and maintained at 25 °C. The detection wavelength was 316 nm. The flow rate was 1.0 mL/min, and the sample injection volume was ten μL. The mobile phase was composed of 0.2% acetonitrile (A) and 1% acetic acid (B), with the following optimized gradient elution: 0–15 min, 62% B; 15–20 min, 62–30% B; 20–40 min, 30% B.

Ferulic acid and ligustilide were quantified through the utilization of external standards. The mixed reference standards of ferulic acid and ligustilide were accurately weighed and dissolved in methanol to achieve final concentrations of 0.006 mg/mL and 0.294 mg/mL for ferulic acid and ligustilide, respectively. The mixed reference standards of ferulic acid and ligustilide were sampled precisely at volumes of 2 μL, 4 μL, 6 μL, 8 μL, 10 μL, 20 μL, 30 μL, and 45 μL and subjected to HPLC analysis.

### 3.9. ATR-FTIR Spectroscopy

The ATR-FTIR spectra of materials were obtained by the Vertex 70 FT-IR/MIR spectrometer equipped with a platinum ATR accessory (Bruker, Mannheim, Germany). Each spectrum had an average of 32 scans in the 4000–400 cm^−1^ range. The influence of water vapor and carbon dioxide was subtracted automatically.

The software Spectrum v10.5 (PerkinElmer, Waltham, MA, USA) was utilized for the spectral analysis. The original ATR-FTIR spectrum variable interval was 1 cm^−1^ and unchanged, whereas the spectral ordinate was transformed into absorbance. Then, the ATR correction with a zero-contact factor and the automatic baseline correction were applied successively. Finally, the spectral ordinate was normalized to ensure that the maximum absorbance value was set to 1 and the minimum absorbance value was set to 0.

### 3.10. Statistical Analysis

All measurements were performed using three biological replicates. The calibration curve for the target ingredients is presented in Table S2. Statistical analysis was performed with ANOVA and Duncan’s multiple comparison tests. The software SPSS version 25.0 (Palo Alto, CA, USA) was used. The statistical significance of the differences was determined based on a subset analysis, where subsets were considered different if *p* < 0.05. On the contrary, the subsets were the same, and there was no significant difference. Prism v8.0 (GraphPad, San Diego, CA, USA) was used for related chart production. Unsupervised pattern recognition techniques, specifically PCA, were employed as an initial approach to investigate variations among the harvest root samples. PLS-DA and OPLS-DA were subsequently modified to conduct discrimination studies. All chemometric classification models were established using SIMCA-P14.1 software (Umetrics A and B, Umea, Sweden). The accuracy of the fitting model was explained by the modeling parameters in the permutation, precisely the values of R^2^ and Q^2^.

## 4. Conclusions

Based on the observations mentioned above, it can be concluded that premature bolting underground in *A. sinensis* leads to a decrease in the accumulation of nutrient components and bioactive metabolites, such as ferulic acid and flavonoids, during the vegetative growth stage. The process of root lignification accompanies this. The occurrence of bolting at an earlier stage results in a significant disparity in the morphological appearance and efficacy components between the unbolted and bolted roots. The utilization of summer as a form of herbal medicine is not feasible. The autumnal root exhibited a composition that consisted of specific bioactive compounds. The substance in question can be utilized as an herbal remedy, health supplement, or infusion for consumption. In the spectral range of 1200–800 cm−1, distinct variations in the characteristic absorption peaks can be observed in the ATR-FTIR spectrum of *A. sinensis* root at different bolting durations. The results were assessed using various statistical techniques, including Spearman’s correlation, PCA, PLS-DA, and OPLS-DA, whereby the ATR-FTIR data demonstrated the most effective outcomes in differentiating between viable and non-viable roots for their application in herbal medicine. The employed method is considered safe, qualitative, and non-destructive to differentiate between samples with varying bolting times. This approach is crucial for ensuring the quality, safety, and efficacy of *A. sinensis*. Essential compounds found in AB roots include ferulic acid and flavonoids, which are significantly more prevalent. The study offers a material foundation for understanding the pharmacological effects of the AB roots and provides a theoretical framework for enhancing their accessibility.

## Data Availability

Not applicable.

## Supplementary Materials

The following supporting information can be downloaded at: https://www.mdpi.com/article/10.3390/molecules28217292/s1.
