# Supplementary material for: The Dynamic Accumulation Rules of Chemical Components during the Medicine Formation Period of Angelica sinensis and Chemometric Classifying Analysis for Different Bolting Times Using ATR-FTIR
Source: Molecules. 2023 Oct 27;28(21):7292. doi: 10.3390/molecules28217292 (PMC10649412; doi:10.3390/molecules28217292)
Supplement: Supplementary file 1 [file molecules-28-07292-s001.zip › molecules-2649046-supplementary.pdf]

Supplementary Materials

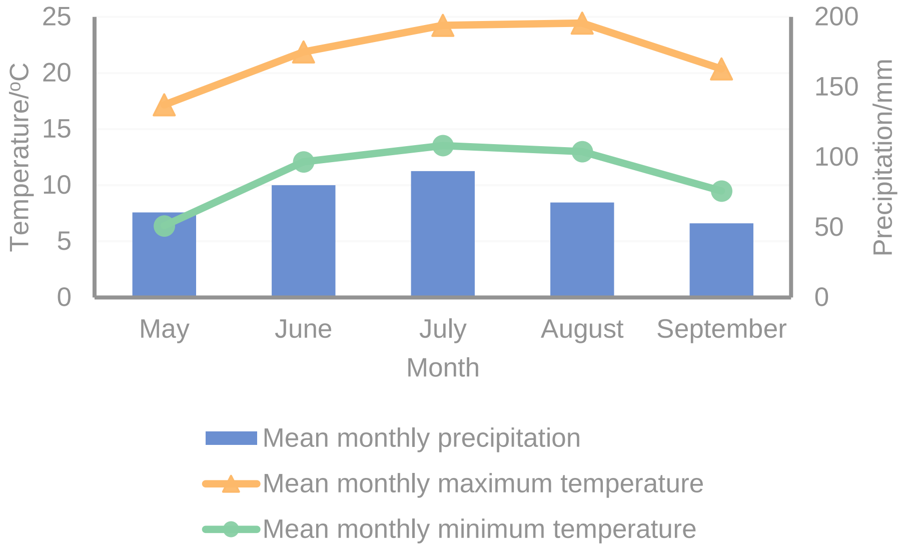

Figure S1. The variation of monthly average temperature and average precipitation in 2019

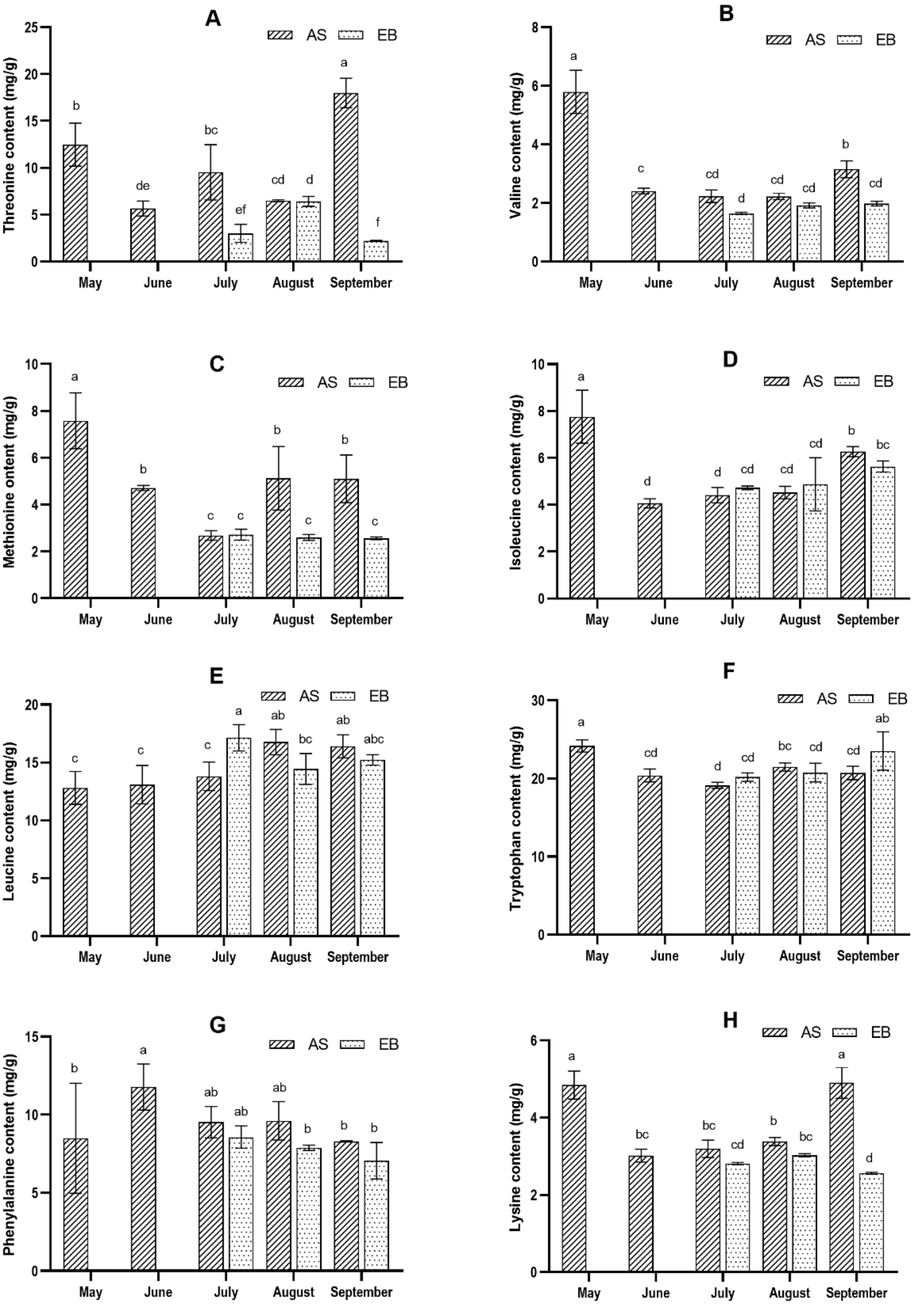

Figure S2. Change in the essential amino acid content of *A. sinensis* in different months: (A) Threonine, (B) Valine, (C) Methionine, (D) Isoleucine, (E) Leucine, (F) Tryptophan, (G) Phenylalanine, (H) Lysine.

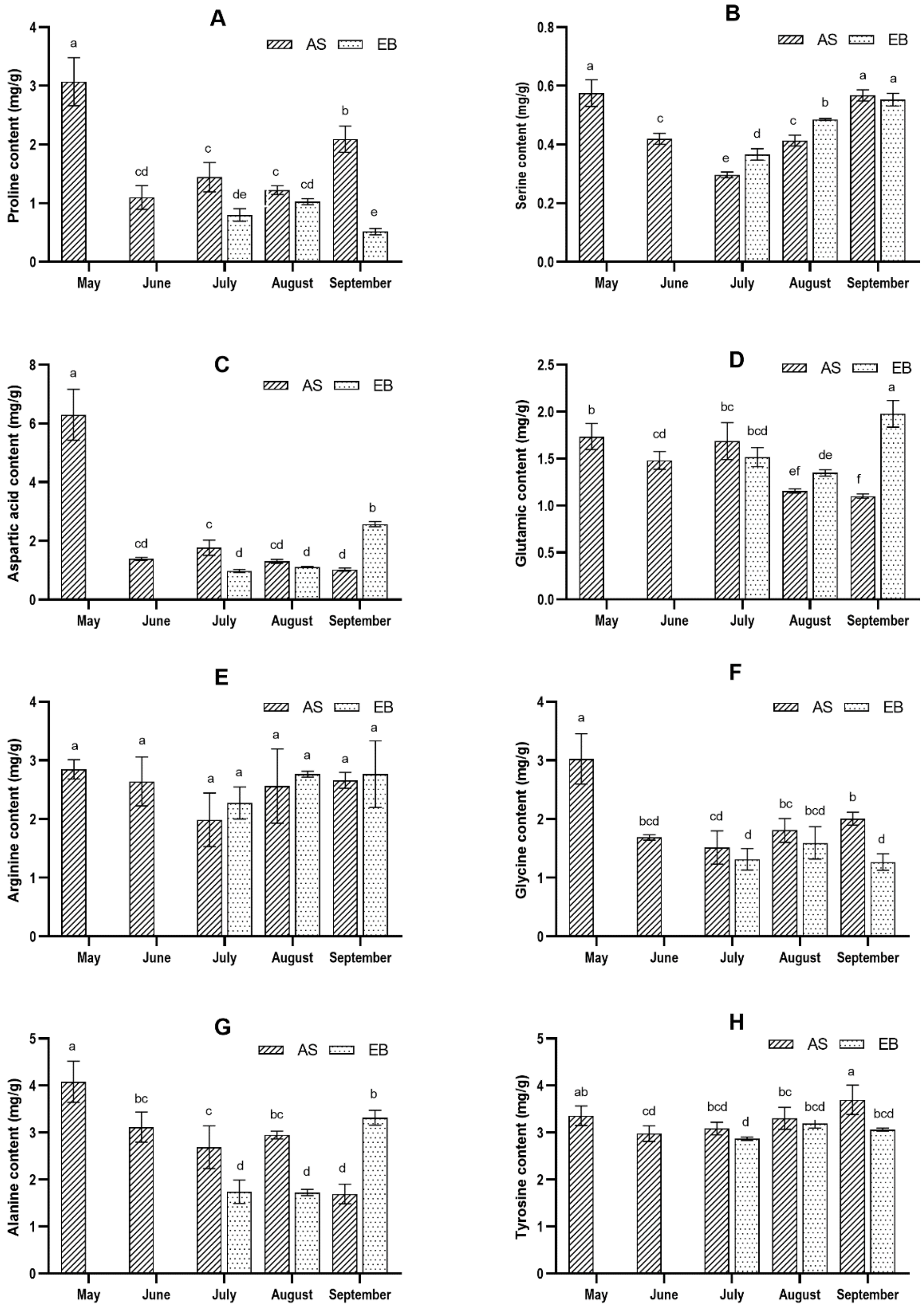

Figure S3. Change in the non-essential amino acid content of *A. sinensis* in different months: (A) Proline, (B) Serine, (C) Aspartic acid, (D) Glutamic, (E) Arginine, (F) Glycine, (G) Alanine, (H) Tyrosine.

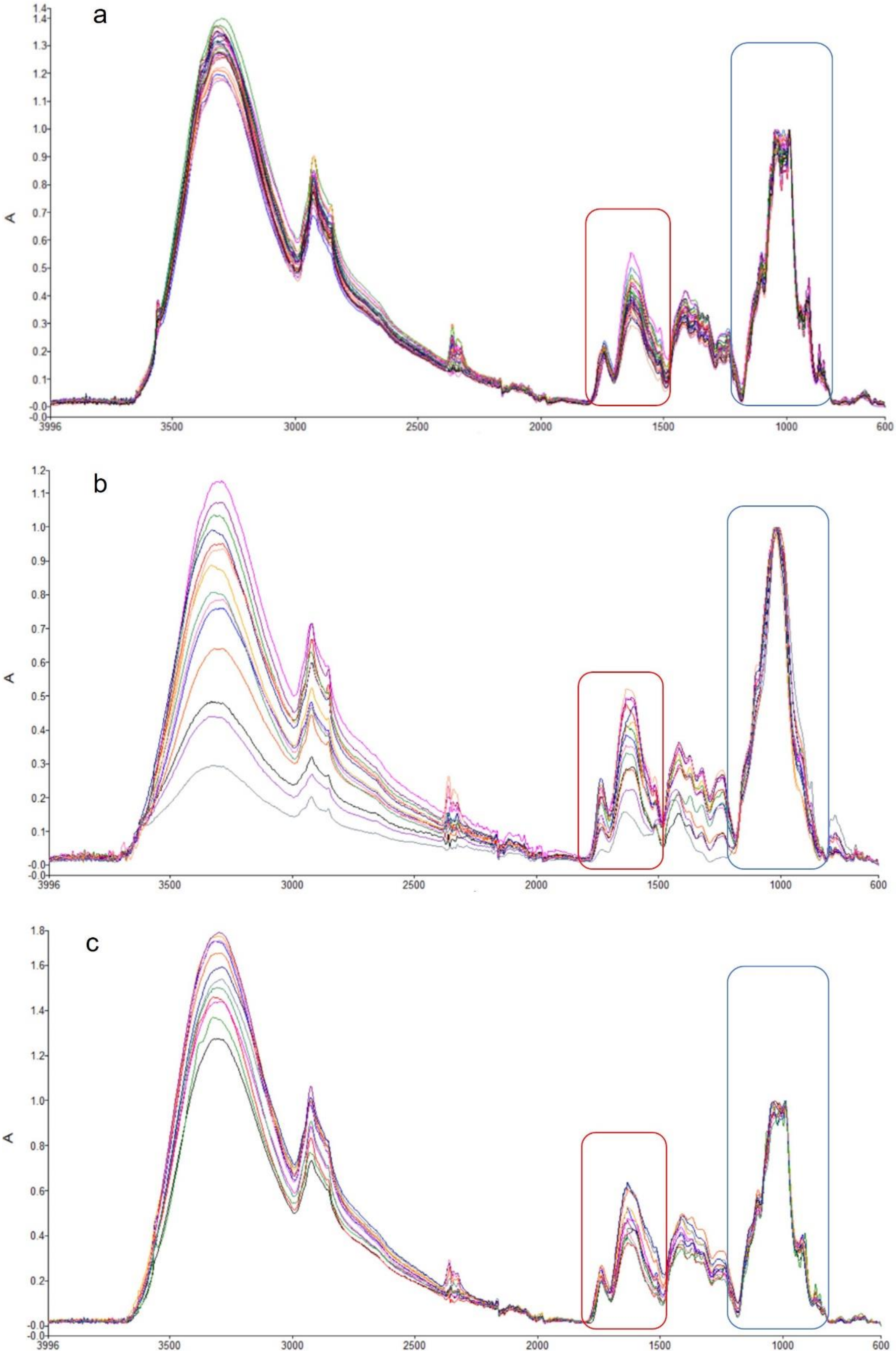

Figure S4. Overall ATR-FTIR spectra of different bolting times in the harvest period: (A) AB root samples, (B) SB root samples and (C) AB root samples

Table S1. Samples information of *A. sinensis* during the root harvest period

Sample	Producing areas	Root length/cm	Root weight/g	Ferulic acid/%	Ligustilide/%	Lignin/%
AS-1	Suigu Village, Min County, Dingxi City	22.30<br>22.10	115.34<br>91.04	0.147	2.474	24.922
AS-2	Suigu Village, Min County, Dingxi City	21.70<br>16.70	37.64<br>13.67	0.180	3.913	0.260
AS-3	Mazichuan village, Min County, Dingxi City	22.10<br>18.30	132.03<br>101.79	0.153	2.947	0.264
AS-4	Duona Village, Min County, Dingxi City	19.50<br>16.80<br>16.50	83.66<br>81.72<br>111.22	0.137	3.182	0.269
AS-5	Duona Village, Min County, Dingxi City	20.20<br>12.00	76.53<br>70.21	0.149	2.053	0.232
AS-6	Shuguang Village, Min County, Dingxi City	8.70<br>5.70<br>15.40<br>7.60	18.06<br>21.05<br>41.74<br>17.87	0.137	2.974	0.245
AS-7	Lizhu Village, Min County, Dingxi City	12.40	141.90	0.184	2.357	0.210

		14.60	72.50			
		15.00	25.37			
AS-8	Lizhu Village, Min County, Dingxi City	10.60	8.98	0.138	3.082	0.260
		13.20	20.06			
AS-9	Qiaojiagou Village, Min County, Dingxi City	13.10	78.01	0.217	4.164	0.257
		12.70	73.88			
AS-10	Qiaojiagou Village, Min County, Dingxi City	16.70	45.03	0.173	4.663	0.255
		14.10	17.09			
		16.40	85.32			
AS-11	Caotan Village, Min County, Dingxi City	16.30	69.09	0.173	3.603	0.216
		19.00	91.45			
AS-12	Caotan Village, Min County, Dingxi City	18.10	38.52	0.172	2.156	0.239
		16.50	41.54			
		19.20	41.14			
AS-13	Anjiashan Village, Min County, Dingxi City	11.80	37.71	0.158	2.933	0.242
		15.70	30.55			
AS-14	Linha Village, Min County, Dingxi City	20.20	135.44	0.171	4.013	0.257
		16.40	25.57			
AS-15	Linha Village, Min County, Dingxi City	18.20	52.63	0.180	3.264	0.244
		14.80	5.74			

AS-16	Wuna Village, Min County, Dingxi City	19.20<br>16.40	86.13<br>70.08	0.154	2.211	0.230
AS-17	Dongtan Village, Tianzhu County, Wuwei City	19.80<br>21.60	56.57<br>36.14	0.170	2.346	0.227
AS-18	Heigou Village, Tianzhu County, Wuwei City	11.20<br>19.40	8.06<br>39.91	0.144	2.049	0.238
AS-19	Heyan Village, Tianzhu County, Wuwei City	17.70<br>23.30	159.64<br>115.44	0.129	2.538	0.223
AS-20	Jianshan Village, Tianzhu County, Wuwei City	21.80<br>15.40	112.96<br>97.02	0.217	2.721	0.233
AS-21	Shangquantan Village, Tianzhu County, Wuwei City	19.30<br>18.70	44.95<br>65.20	0.178	3.738	0.252
AS-22	Huangjiading Village, Linzhao County, Dingxi City	14.50<br>17.50	82.61<br>47.31	0.125	4.723	0.246
AS-23	Huangjiading Village, Linzhao County, Dingxi City	13.20<br>16.50<br>12.00<br>13.30<br>12.10	6.13<br>1.26<br>2.15<br>0.76<br>10.35	0.128	3.656	0.228
AS-24	Xialuo Village, Dangchang County, Longnan City	18.60	92.26	0.184	2.721	0.233

		17.20	11.92			
		16.60	22.60			
		16.00	13.73			
		16.90	18.61			
		20.00	113.12			
		17.10	80.60			
AS-25	Majie Village, Dangchang County, Longnan City	16.20	87.52	0.208	2.489	0.266
		13.50	32.33			
		20.90	59.23			
		17.60	53.93			
AS-26	Gufeng Village, Gulang County, Wuwei City	12.10	73.10	0.163	1.988	0.261
		20.00	74.67			
AS-27	Yangjiasi Village, Weiyuan County, Dingxi City	22.50	46.87	0.249	5.857	0.253
		18.50	14.25			
		17.50	37.39			
AS-28	Jianshan Village, Weiyuan County, Dingxi City	13.30	41.44	0.201	4.810	0.237
		11.10	15.42			
		10.10	13.56			
AS-29	Benmiao Village, Huichuan County, Dingxi City	12.60	21.74	0.165	3.015	0.229
		8.80	4.27			

		14.80	5.88			
		15.50	21.53			
		13.40	7.07			
		11.00	19.30			
		9.30	5.38			
SB-1	Mazichuan Village, Min County, Dingxi City	7.40	5.72	0.112	1.103	0.292
		8.70	3.63			
		5.90	2.68			
		7.20	2.93			
SB-2	Suigu Village, Min County, Dingxi City	11.70	19.69	0.057	1.340	0.261
		16.50	6.74			
		16.50	7.06			
SB-3	Lizhu Village, Min County, Dingxi City	16.00	9.16	0.105	1.130	0.266
		8.70	3.57			
SB-4	Caotan Village, Min County, Dingxi City	13.00	20.24	0.053	0.690	0.291
		16.60	13.50			
		13.50	7.55			
SB-5	Heyan Village, Tianzhu County, Wuwei City	11.10	12.48	0.014	0.514	0.273
		9.80	4.68			
		10.00	3.52			

		12.00	9.05			
		9.80	5.75			
		12.50	6.30			
		6.90	14.49			
SB-6	Jianshan Village, Tianzhu County, Wuwei City	11.60	50.81	0.071	0.967	0.239
		8.90	10.40			
SB-7	Shangquantan Village, Tianzhu County, Wuwei City	16.90	7.28	0.069	1.033	0.308
		13.50	6.58			
		8.50	12.27			
		9.00	5.56			
SB-8	Huangjiading Village, Linzhao County, Dingxi City	5.00	4.41	0.130	1.149	0.273
		6.70	2.07			
		10.50	2.01			
SB-9	Majie Village, Dangchang County, Longnan City	9.10	9.66	0.080	0.877	0.298
		9.00	6.59			
SB-10	Xialuo Village, Dangchang County, Longnan City	6.30	12.52	0.076	0.721	0.272
		9.50	14.70			
SB-11	Gufeng Village, Gulang County, Wuwei City	10.4	6.02	0.045	0.670	0.285
		8.40	23.38			
SB-12	Jianshan Village, Tianzhu County, Wuwei City	9.40	9.77	0.097	1.154	0.218

		11.60	43.10			
SB-13	Yangjiasi Village, Weiyuan County, Dingxi City	8.70	3.55	0.090	0.981	0.350
		6.10	4.25			
SB-14	Benmiao Village, Huichuan County, Dingxi City	4.40	12.86	0.014	0.551	0.233
		5.40	6.50			
AB-1	Mazichuan Village, Min County, Dingxi City	13.60	123.40	0.168	1.934	0.266
		15.80	87.74			
		17.60	78.14			
AB-2	Duona Village, Min County, Dingxi City	18.70	78.39	0.179	1.540	0.249
		15.80	45.07			
		18.40	98.40			
		15.70	29.66			
AB-3	Wuna Village, Min County, Dingxi City	18.30	22.52	0.143	1.380	0.253
		19.30	30.29			
		18.70	81.60			
		14.00	97.73			
AB-4	Liha Village, Min County, Dingxi City	9.80	45.70	0.092	1.804	0.238
		6.90	26.74			
AB-5	Heigou Village, Tianzhu County, Wuwei City	13.00	20.69	0.130	0.876	0.242
		12.30	8.40			

AB-6	Dongtan Village, Tianzhu County, Wuwei City	10.00	17.81	0.116	1.226	0.250
		24.10	19.53			
AB-7	Huangjiading Village, Linzhao County, Dingxi City	13.50	72.84	0.239	2.537	0.262
		11.50	50.10			
AB-8	Majie Village, Dangchang County, Longnan City	12.40	47.81	0.209	1.741	0.245
		13.30	34.38			
		11.70	16.99			
AB-9	Xialuo Village, Dangchang County, Longnan City	18.60	105.52	0.185	3.241	0.253
		10.40	63.39			
AB-10	Gufeng Village, Gulang County, Wuwei City	19.10	53.29	0.107	1.251	0.267
		23.10	62.73			
AB-11	Yangjiasi Village, Weiyuan County, Dingxi City	14.70	38.33	0.108	1.324	0.241
		13.50	14.60			
		5.50	14.11			
AB-12	Benmiao Village, Huichuan County, Dingxi City	11.50	45.32	0.231	2.586	0.292
		9.50	30.64			

Table S2. The calibration curve of target ingredients

Ingredients	Calibration curves	Related coefficient (R^2)
Glucose	y= 8.3224x+0.0238	0.9957
Bovine serum albumin	y= 1.0328x - 0.0113	0.9978
Aspartic acid	y = 794771695.1x - 5154.18264	0.9999
Glutamic	y = 689728928.5x - 4925.76946	0.9999
Serine	y = 11031886054.0x - 225339.42860	0.9949
Arginine	y = 948120475.5x - 1577.70817	0.9925
Glycine	y = 799568974.6x - 5546.63174	0.9998
Threonine	y = 620208409.3x + 8122.52994	0.9988
Proline	y = 122714429.8x - 2866.33533	0.9955
Alanine	y = 1793349036.0x - 19172.81437	0.9998
Valine	y = 595828728.9x - 5823.73653	0.9989
Methionine	y = 662691616.8x - 4509.14970	0.9991
Isoleucine	y = 299348470.1x - 8414.06226	0.9965
Leucine	y = 912231372.5x - 546.07843	0.9984
Tryptophan	y = 1021649334.0x - 43539.47605	0.9981
Phenylalanine	y = 445585846.3x + 2892.64981	0.9932
Lysin	y = 979015908.2x - 22262.83234	0.9999
Tyrosine	y = 588521850.2x - 15652.05988	0.9997
Ferulic acid	y = 5032691.1010x - 297.1657	0.9999
Ligustilide	y= 516197.0035x	0.9999
Lignin	y = 28.707x + 0.1724	0.9921
Total C element	y = 8923.00529x - 316.3809x^2	0.9999
Total N element	y = 3231.03527x	0.9998
